# IBVis: Interactive Visual Analytics for Information Bottleneck Based Trajectory Clustering

**DOI:** 10.3390/e20030159

**Published:** 2018-03-02

**Authors:** Yuejun Guo, Qing Xu, Mateu Sbert

**Affiliations:** 1 School of Computer Science and Technology, Tianjin University, Tianjin 300350, China; 2 Department of Informàtica i Matemàtica Aplicada, University of Girona, 17071 Girona, Spain

**Keywords:** visual analytics, information bottleneck, trajectory clustering

## Abstract

Analyzing trajectory data plays an important role in practical applications, and clustering is one of the most widely used techniques for this task. The clustering approach based on information bottleneck (IB) principle has shown its effectiveness for trajectory data, in which a predefined number of the clusters and an explicit distance measure between trajectories are not required. However, presenting directly the final results of IB clustering gives no clear idea of both trajectory data and clustering process. Visual analytics actually provides a powerful methodology to address this issue. In this paper, we present an interactive visual analytics prototype called IBVis to supply an expressive investigation of IB-based trajectory clustering. IBVis provides various views to graphically present the key components of IB and the current clustering results. Rich user interactions drive different views work together, so as to monitor and steer the clustering procedure and to refine the results. In this way, insights on how to make better use of IB for different featured trajectory data can be gained for users, leading to better analyzing and understanding trajectory data. The applicability of IBVis has been evidenced in usage scenarios. In addition, the conducted user study shows IBVis is well designed and helpful for users.

## 1. Introduction

In general, a trajectory is the path generated by a moving object (for example, airplane, animal and pedestrian) and is recorded as a sequence of sample points. Each sample point, happening at a certain time, includes many possible attributes of the object, such as location, direction/angle, speed and so on. Due to the rapid prevalence and development of sensor systems, large amounts of trajectory data are collected in a lot of applications, for instance, intelligent transportation system [1], animals’ migratory analysis [2] and network intrusion detection [3]. Undoubtedly, the analysis and understanding of these data are very important.

Clustering is one of the most important techniques to help grasp the underlying nature of complex trajectory data, and has become a hot topic [4]. Given a trajectory dataset, clustering algorithms aim to partition the trajectories into different groups such that each trajectory is most similar to the others within the same group, and that trajectories belonging to different groups are most dissimilar. For many clustering algorithms proposed so far, it is necessary to specify a distance measure to calculate the similarity between trajectories. However, as indicated by [5,6], the distance measures either result in diverse accuracies with different data sizes or resort to parameters, which means that obtaining a very general and good measure is non-trivial for dealing with various kinds of trajectory data. In addition, the pre-determination for either the number of clusters or the termination threshold, and even for both, has to be done for most clustering algorithms, such as *k*-means [7] and hierarchical clustering [8]. For the purpose of this paper, the information bottleneck (IB) based clustering, which has been demonstrated as an effective approach to manage the issues mentioned above [9,10,11], is utilized for trajectory data. Actually, the IB principle, which is an information theoretic approach, has been applied in many fields [12], such as image segmentation, document clustering and so on.

In general, to show the performance of a clustering algorithm, only the final clusters and their corresponding quantitative evaluations are presented. Obviously, simply checking and analyzing the results from performing IB-based clustering on different trajectory datasets help demonstrate the effectiveness of IB, but this gives little insight to thoroughly understanding the mechanism of IB on how to work on trajectory data. To deal with this issue, we take advantage of the visual analytics methodology, which has attracted increasing attention in the field of trajectory analysis [4]. A lot of visual analytics approaches related to trajectory analysis have been proposed [13,14], although most of them solely involve the visualization of trajectory attributes and analysis results. In this case, it is not easy for users to understand the algorithmic procedure of the analysis method.

In this paper, we propose a visualization system, IBVis, providing users with rich interactions to support the analysis of IB-based trajectory clustering. IBVis offers two major modules, Clusters Generation and Results Comparison, which interact with each other and focus on the clustering procedure and cluster results respectively. The module of Clusters Generation includes four views, namely, Dashboard, Trajectory, Merging and Backtracking and Plot, comprehensively visualizing the clustering procedure. The module of Results Comparison gives information of parameter setting and performance evaluation for different clustering results. Users can choose their specified parameters to check and compare the ranking of the corresponding outputs, which helps to construct ideas on how to make better use of IB to obtain high quality clustering results. The main contributions of this paper are as follows:

1. IBVis, a novel visualization system, is designed to clearly interpret the IB-based clustering procedure and to present its outcomes.
2. IBVis offers comprehensive interactions for users to achieve a deep and complete understanding of the mechanism of IB. For instance, users can backtrack the clustering procedure after finishing all the iterations, and arrange a different merging in a certain iteration to fulfill a better clustering.
3. IBVis provides a friendly interface to guide users to walk through the system. This is especially helpful for users which are unfamiliar with trajectory analysis.
4. Finally, we conduct experiments of the usage scenarios and a user study to explain and evaluate the usefulness of IBVis.

The rest of this paper is organized as follows: Section 2 covers the related work; Section 3 explains the IB-based trajectory clustering; Section 4 is a system overview of IBVis; The two major components of IBVis, Clusters Generation and Results Comparison, are described clearly in Section 5 and Section 6, respectively. Section 7 gives the usage scenarios of IBVis; We conduct a user study on IBVis in Section 8; Finally, concluding remarks and future work are given in Section 9.

## 2. Related Work

Clustering, as a typical analysis technique, has been extensively studied to discover massive meaningful information from trajectory data [4]. For instance, *k*-means is one of the most widely used algorithms due to its simplicity and computational efficiency [7,15]. To perform *k*-means, users need to specify the number of clusters *k*, the initial *k* centers of clusters, and a distance measure to compute the similarity between trajectories. In each iteration, the center of a cluster is selected to minimize the summed distances between it and all the other trajectories in this cluster. After some iterations, trajectories are grouped into *k* clusters until each trajectory has the smallest distance with the center of its belonging cluster. For trajectory clustering, there exist many additional classical approaches applied such as BIRCH (Balanced Iterative Reducing and Clustering using Hierarchies) [16] and DBSCAN (Density-Based Spatial Clustering of Applications with Noise) [17], the state-of-the-art methods such as DPMM (Dirichlet process mixture model) [18,19] and partition-and-summarization framework [20]. However, most clustering techniques have the issues that they resort to some predefined parameters, such as the number of clusters and the similarity threshold, in practice. In this paper, the information bottleneck (IB) principle [21] is utilized in the proposed visual analytics prototype for trajectory clustering, so that the issues aforementioned are managed.

The visual analytics methodology has been popularly developed in many fields to facilitate experts and end-users to solve complex problems. For instance, WEKA [22] and RapidMiner [23] are two comprehensive softwares which include a collection of machine learning algorithms and visualization tools. Furthermore, these software platforms are well-suited for users to develop new algorithms. Löwe et al. design a visual analytics framework [24] for the domain experts to make a proper decision on the order selection criteria of an certain autoregressive process. The SmartAdP system obtained by Liu et al. gives an optimal billboard location based on different views [25]. To know more about visual analytics, please refer to recent relevant literatures [26,27,28].

Generally, in the field of trajectory clustering, most existing visualization tools mainly focus on visualizing and interpreting the clustering outputs, and users cannot manipulate the clustering process and its results. For example, Turkay et al. introduce two methods to vividly display the temporal cluster structures [13]. Chen et al. make use of the widely accepted visual clustering method SOM (self-organizing map) on bankruptcy trajectory data to mine their patterns for financial analysis [14], however, the public gets little understanding of the methodology of SOM. Munaga et al. propose a visualization tool named CAST (Clustering And visualization tool for Spatio-Temporal data) [29] for effectively exploring the trajectory data. Another similar visualization tool named DenTrac (Density based Trajectory Clustering and visualization tool for Spatio-Temporal data) [30] is designed to have the same function with CAST, and the only difference between them is the clustering algorithm applied. Andrienko et al. extend the concept of STC (Space-time cube) [31] with different transformations on time [32], leading to a better display of the dynamics within clusters.

Interactive manipulations on the procedure of clustering algorithm, together with (graphical) presentations on the relevant clustering results, contribute a lot to the deep analytics and understanding of the algorithm. Chang et al. present multi-granularity views of the clustering results by allowing users to set the density of clusters [33]. By observing results in different levels of granularities, users can obtain both the overview and detailed knowledge of large trajectory data. However, due to the lack of quantitative performance evaluations, it is difficult to know whether the clusterings based on various cluster densities works well or not. WEKA [22] has implemented the sequential IB clustering, which is different from the agglomerative IB used in IBVis. Both the agglomerative IB and the sequential IB are derived from the IB principle [12] but have different strategies to perform the clustering. The agglomerative IB adopts a bottom-up and iterative strategy to obtain the clusters. Taking trajectory clustering as an example, each trajectory initially is assigned to a singleton cluster, and then two similar clusters are combined in each iteration. The sequential IB takes a “sequential *k*-means like” strategy. Initially all the trajectories are assigned into a certain number of clusters. Then at each step, every trajectory is visited and re-assigned to a proper cluster. Fan et al. have recently attempted to design a 3D visualization tool for agglomerative IB clustering [34], which has shown its potential in helping users study and understand IB. However, this visualization tool pays much attention to depict the conditional probabilities used for performing the clustering, but fails to present the running iterations of IB. Our proposed IBVis is well designed to cope with the issues mentioned above. Through different views, users can understand on trajectory data and IB very well. In particular, the iteration procedure can be monitored and steered to gain insight on how to improve IB.

## 3. Trajectory Clustering Based on IB

In this section, we briefly review the information bottleneck (IB) principle for the clustering of trajectory data, for more details please refer to [9].

The information bottleneck principle was proposed by Tishby et al. in 1999 [21], aiming at partitioning a dataset into several clusters by using a corresponding feature set. Given a trajectory dataset *X* and its feature set *Y*, a set of trajectory clusters $\tilde{X}$ can be achieved by maximizing the shared information between $\tilde{X}$ and *Y*,

(1) $$L_{max}=\left\{I\left(\tilde{X};Y\right)\right\},$$

where $I\left(\tilde{X};Y\right)$ is the mutual information defined in the information theory tools [21]. Here trajectory attributes, such as location, shape, speed of the moving object and so on, can be used for *Y*. Practically, a simple but effective clustering, called agglomerative information bottleneck [12,35], is organized in a bottom-up and iterative manner to merge elements in *X* to get the final clusters. Initially *X* is copied as the initial $\tilde{X}\left({\tilde{X}}_{0}\right)$, here each trajectory $x_{i}\in X$, corresponding to a single cluster, behaves as an element in ${\tilde{X}}_{0}$, ${\tilde{X}}_{0}=\left\{\left\{x_{i}\right\}\right\}$. In each iteration, two elements of the current hierarchy $\tilde{X}\left({\tilde{X}}_{h}\right)$ are merged to obtain the next hierarchical $\tilde{X}\left({\tilde{X}}_{h+1}\right)$, meeting the minimization of the loss of mutual information $\Delta L_{max}=L_{max}^{b}-L_{max}^{a}$, where $L_{max}^{b}=I\left({\tilde{X}}_{h};Y\right)$ and $L_{max}^{a}=I\left({\tilde{X}}_{h+1};Y\right)$ respectively represent the mutual information before and after the merging. Notice that the merging procedure fulfills the maximization of Equation (1) in an iterative manner.

The computation of agglomerative information bottleneck [12,35] is detailed as follows. Suppose ${\tilde{x}}_{1}$ and ${\tilde{x}}_{2}$ are the two candidate clusters for merging in a certain iteration, ${\tilde{x}}^{*}$ is the new cluster produced by this merge, and the loss of mutual information caused is deduced as [35]

(2) $$\begin{array}{cc} \;\Delta L_{max}\left({\tilde{x}}_{1},{\tilde{x}}_{2}\right) & =p\left({\tilde{x}}^{*}\right)JSD\left(\frac{p\left({\tilde{x}}_{1}\right)}{p\left({\tilde{x}}^{*}\right)},\frac{p\left({\tilde{x}}_{2}\right)}{p\left({\tilde{x}}^{*}\right)};p\left(Y|{\tilde{x}}_{1}\right),p\left(Y|{\tilde{x}}_{2}\right)\right). \end{array}$$

Here JSD indicates the Jensen-Shannon divergence [36], which is a basic metric for the difference between two probabilities $P_{1}$ and $P_{2}$ with two positive weights $w_{1}$ and $w_{2}$

(3) $$JSD_{w_{1},w_{2}}\left(P_{1},P_{2}\right)=H\left(\sum_{i=1,2}w_{i}P_{i}\right)-\sum_{i=1,2}w_{i}H\left(P_{i}\right),$$

where $H\left(P\right)=\sum_{p_{i}\in P}p_{i}\log p_{i}$ is the Shannon entropy of a probability distribution *P*. Notice that the introduction of JSD eases the calculation of the loss of mutual information needed in the clustering. The prior and conditional probabilities of ${\tilde{x}}^{*}$ are obtained by

(4) $$p\left({\tilde{x}}^{*}\right)=p\left({\tilde{x}}_{1}\right)+p\left({\tilde{x}}_{2}\right)$$

and

(5) $$p\left(y_{j}|{\tilde{x}}^{*}\right)=\frac{p\left({\tilde{x}}_{1}\right)}{p\left({\tilde{x}}^{*}\right)}p\left(y_{j}|{\tilde{x}}_{1}\right)+\frac{p\left({\tilde{x}}_{2}\right)}{p\left({\tilde{x}}^{*}\right)}p\left(y_{j}|{\tilde{x}}_{2}\right),$$

in practice $Y=\{y_{j}\}$ and $p\left(Y|x\right)=\left\{p\left(y_{j}|x\right)\right\}$ (j=1,2,…,n) are respectively a discrete feature set and a discrete probability distribution function (PDF) obtained by the kernel density estimation (KDE) modeling, and *n* is the number of discrete probability bins [9]. In the beginning of clustering, $p\left({\tilde{x}}_{1}\right)=p\left({\tilde{x}}_{2}\right)=\frac{1}{\left|X\right|}$, and here ${\tilde{x}}_{1}$ and ${\tilde{x}}_{2}$ correspond to two single trajectories in *X*.

IB-based trajectory clustering algorithm tries to find a number of clusters which group the similar trajectories together, and at the same time, to preserve the information related to the feature set. Thus, the ratio of mutual information between the clustering result and feature set to the one between the original data and feature set, $\delta_{MI}=\frac{I\left(\tilde{X};Y\right)}{I\left(X;Y\right)}$, can be used to adaptively terminate the iterative process. IB performs good outputs with $\delta_{MI}\geq 95\%$.

## 4. An Overview of IBVis

**Figure 1:**
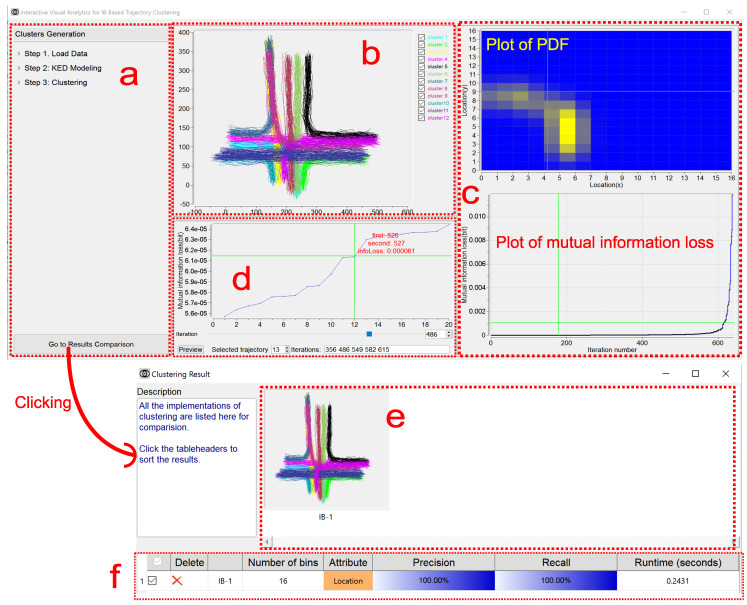
An overview of IBVis. The CROSS (see Section 7) dataset is used. (1) Interface of Clusters Generation (upper), (**a**) Dashboard View, (**b**) Trajectory View, (**c**) Plot View, (**d**) Merging and Backtracking View; (2) Interface of Results Comparison (bottom), (**e**) Clusters View, (**f**) Ranking View.

In this section, we present our interactive visualization prototype, IBVis, which is effective to graphically present and help understand the IB-based trajectory clustering. Figure 1 shows an overview of IBVis.

IBVis includes two modules, Clusters Generation and Results Comparison. The module Clusters Generation is elaborately designed to deeply comprehend and analyze IB, and this module is divided into four views, namely, Dashboard View, Trajectory View, Merging and Backtracking View and Plot View. The Dashboard View acts as an interface to enable users to adjust the parameters for clustering to navigate its procedure. All the trajectories are rendered in the Trajectory View, and each of them is represented by a series of 2D spatial coordinates (locations) of the sample points. The trajectory being handled is highlighted by color (red, for example). The Merging and Backtracking View allows users to backtrack the iterative procedure. For a certain iteration, this view gives a detailed information of the clusters, facilitating users to specify the clusters for merging. While either the PDF of the trajectory is being modeled or the mutual information loss is being resulted from merging, the corresponding display keeps updating in the Plot View. The module Results Comparison, which makes users know the clustering outputs generated with different settings of parameters, involves Clusters View and Ranking View. Based on the combination of the visual examination of different clustering results shown in Clusters View and the quantitative comparison of the corresponding performance evaluations listed in Ranking View, users can easily recognize good parameters for obtaining satisfactory clustering results and in this case, to propose possible strategies for the improvement of IB.

All the components of IBVis are developed using QT GUI framework (version: 5.8.0). The built-in KDE modeling used by IBVis is implemented based on the powerful KDE toolbox in Matlab (version: R2013a). All the IBVis relevant results in this paper are obtained on a Windows machine with Intel Core i7 2.40 GHZ CPU and 8 GB RAM.

## 5. The Module of Clusters Generation

The window of this module, involving four views, supplies rich graphical representations and interactive manipulations, and helps users understand well the IB-based clustering algorithm. The four embedded views are described in detail below.

### 5.1. Dashboard View

The Dashboard View provides a set of interactive manipulations. Considering that users could be not familiar with the knowledge of IB-based clustering, this view simply gives an intuitive guidance for them to run the algorithm.

**Figure 2:**
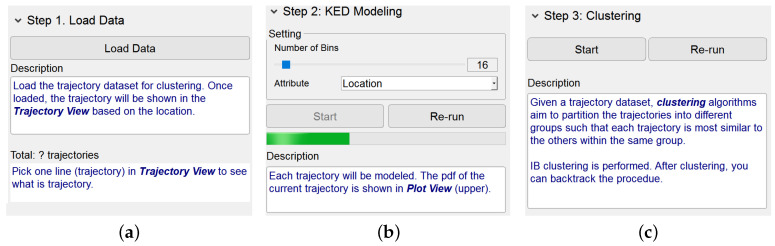
Interface of Dashboard View. (**a**–**c**) show the design and description of three panels, “Load Data”, “KDE Modeling” and “Clustering”, respectively. (**a**) Panel of step 1; (**b**) Panel of step 2; (**c**) Panel of step 3.

Basically, this view supplies three menu items corresponding respectively to the steps of the IB-based clustering algorithm, namely, Load Data, KDE Modeling and Clustering. By clicking on an item, the layout of its functionalities and the relevant descriptions appear. Once a step of the algorithm has been finished, its corresponding layout is hidden. This obviously helps users concentrate on the algorithmic step being performed. Notice that, as shown in Figure 2, the description texts for each step, containing the highlighted keywords, are helpful for users to gain the knowledge of the trajectory clustering based on IB.

The button “Go to Results Comparison” at the bottom of this view enables users to go to the module Results Comparison.

### 5.2. Trajectory View

**Figure 3:**
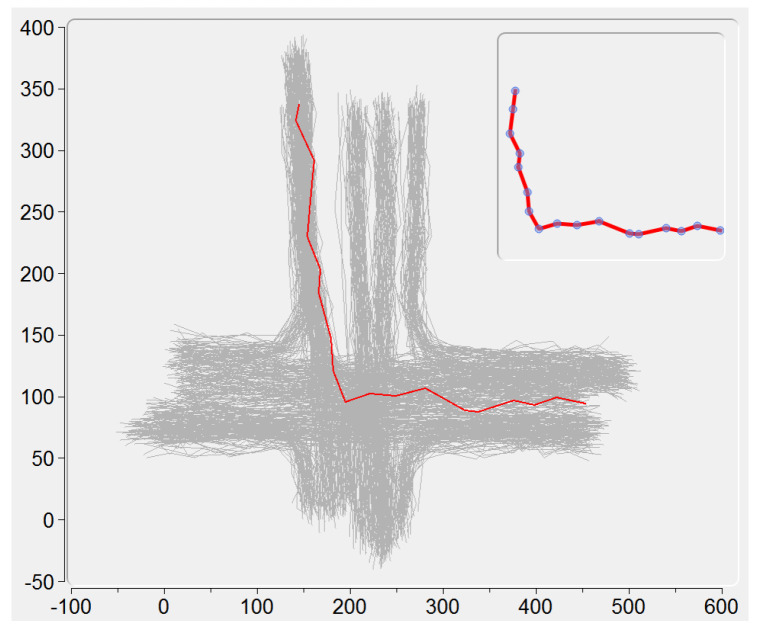
The sample points (in blue color) animate to demonstrate a single trajectory under selection (in red color). The X and Y-Axis represent the horizontal and vertical positions, respectively.

All the loaded trajectories are displayed here by using the locations of their sample points. When clicking on a single trajectory, its sample points animate in a same speed, giving an intuitive demonstration of this trajectory (see an example in the upper right corner of Figure 3).

In the procedure of KDE modeling, the trajectory being processed is visualized in red color, and all the trajectories that have been modeled are visualized in green color. Via clicking on the trajectory when its modeling has been finished, the PDF of this trajectory is displayed in Plot View.

In the procedure of IB-based clustering, the two trajectory clusters being merged are displayed in red and blue colors respectively. After the clustering procedure has been finished, all the final trajectory clusters are visualized in different colors. In the meantime, the checkboxes corresponding to the final clusters are used to show either all or part of these clusters to avoid the possible clutter of complex clustering.

### 5.3. Plot View

This view includes two kinds of plots for the displaying of PDF and mutual information loss, respectively.

**Figure 4:**
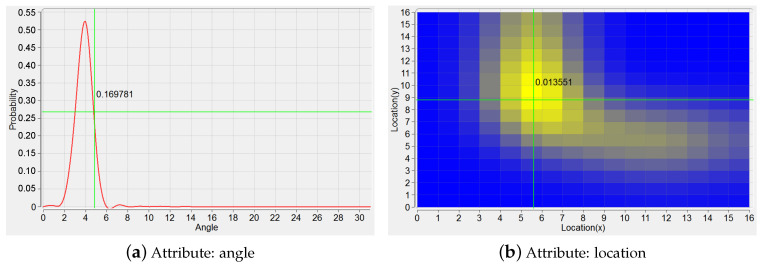
PDFs based on the trajectory attributes. (**a**) KDE modeling based on angle; (**b**) KDE modeling based on location.

The plot of PDF can be represented as a 1D continuous functional curve (Figure 4a) when KDE modeling is based on the shape attribute of a trajectory, here the tangent angles happened at sample points characterize this attribute. If the location attribute is considered for the modeling of a trajectory, the plot of PDF can be depicted vividly in a 2D manner on the ground plane (Figure 4b). That is, the plane is segmented into a gird of a number of spatial cells, and the color of each cell is set according to the corresponding PDF value.

**Figure 5:**
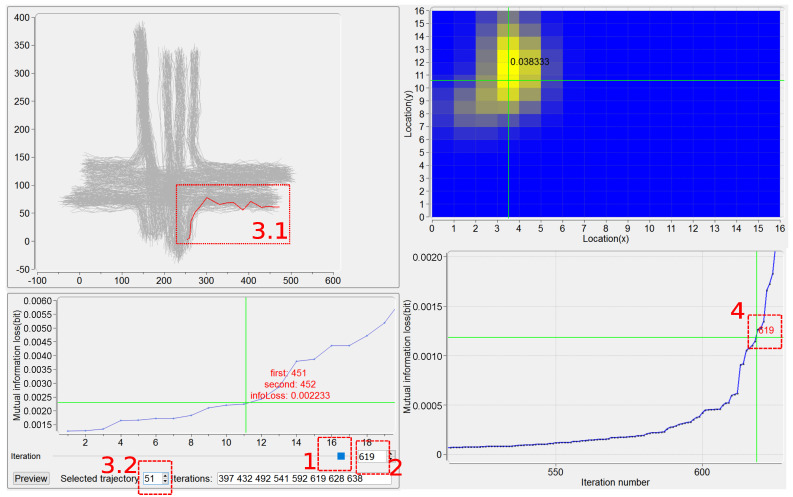
Four ways to locate to a certain iteration. In this example, the 619th iteration is located.

The plot of mutual information loss (Figure 1) keeps updating in the procedure of IB-based clustering, showing the loss value for each iteration. Considering that many iterations could happen due to a large size of the trajectory dataset and that the values of mutual information loss could have a high dynamic range, the zooming in and out is supported for this plot, by the use of mouse, to enable a better understanding of the clustering from both detailed and global perspectives. Furthermore, users can be moved to a specified iteration by the click of its number on the plot, this will be detailed in Section 5.4 and Figure 5.

### 5.4. Merging and Backtracking View

As discussed in our previous work [9], the agglomerative IB, which obtains, for each iteration, a new merged cluster based on the two clusters giving rise to the minimization of the mutual information loss happened in this iteration, may not get a global optimum for the whole clustering. This view is developed as an interactive visual interface to “backtrack” the whole iterative procedure of the clustering, that is, to examine the behavior of any IB iteration and if necessary, to remerge two clusters that are different from before for a certain iteration, alleviating the local optimization issue as much as possible.

This view, as presented in Figure 5, basically supplies, for each iteration, the plot of a number, *n*, of the least mutual information loss caused by pairs of clusters. In the current implementation, n=20 works well for all the trajectory datasets we have tested. As an example in the bottom left part of Figure 5, for iteration number 619, the merging of the 11th pair of clusters (the clusters numbers 451 and 452) results in the mutual information loss 0.002233. Other widgets at the bottom of this view move users to a certain iteration, helping achieve both the behavior examination on this iteration and also the possible remerging operation.

Users can be moved to a certain iteration based on one of the following four kinds of interactions, described as follows. First, the use of the slider (see region 1 in Figure 5) is straightforward, but somewhat arbitrary. Second, the iteration number can be input directly in the textbox (see region 2 in Figure 5). Third, a trajectory is specified either by picking it up in Trajectory View (see region 3.1 in Figure 5) or by filling in its ID number (see region 3.2 in Figure 5), and the relevant iterations in which this trajectory is involved for being merged are identified. For example, the trajectory with ID=51 is specified and the numbers of its relevant iterations, $\left\{397,432,492,541,592,619,628,638\right\}$, are recognized (Figure 5). Notice that in this case, if a specified trajectory is found being incorrectly clustered, then the iteration in which a wrong merging of clusters happens can be easily located. Here the PDF of the specified trajectory is also displayed in Plot View, as a “highlight” on this trajectory. Fourth, an iteration can be addressed by clicking on the plot of the least mutual information loss (see region 4 in Figure 5). Note that, given a certain iteration, all the information corresponding to the four kinds of interactions mentioned above is synchronized.

**Figure 6:**
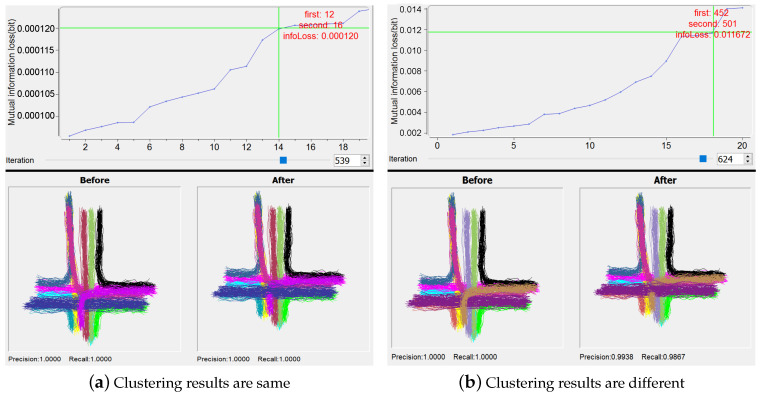
Examples of changing the merging in one iteration. In different iterations, choosing the merging clusters results in same (**a**) or different (**b**) clusterings.

By the manipulation on the plot of the least mutual information loss for a certain iteration, users can investigate the candidate pairs of clusters to determine whether they are acceptable to be merged. The trajectories belonging to two candidate clusters are highlighted in Trajectory View. By the use of button “Preview”, the clustering results due to before and after “manual” merging are visualized in Clusters View, and the corresponding quantitative performance results are given in Ranking View (see the examples in Figure 6; Clusters View and Ranking View will be discussed in Section 6.1 and Section 6.2 respectively).

## 6. The Module of Results Comparison

The window of this module provides two views to present all the clustering results by IB under different settings in graphical and descriptive manners respectively, as shown in Figure 1.

### 6.1. Clusters View

This view simply presents visually a straightforward comparison between different clustering results. Different clusters are visualized in varied colors. It is noted that the contents of this view respond synchronically to interactions, such as deleting (“Delete”), operated in Ranking view.

### 6.2. Ranking View

This view is organized as a table, for the IB clustering, to analyze and understand the relationship between its parameter settings and the corresponding performance results. Each row of the table gives a concise information on a trial of IB clustering. Currently, the parameters (number of discrete probability bins and trajectory attributes) used in KDE modeling and the performance evaluations (precision, recall and runtime) on the clustering are concerned as the information items. Quantitative values and additionally the color bar are used for presenting precision and recall metrics. The higher the metric value is, the more color levels from light to dark present. Each trial of clustering is named by its sequence index, for instance, IB-1 means the first trial. The checkbox offers a way to determine whether a certain trial is involved or not, for the sake of being investigated. All the involved IB trials can be sorted according to which kind of information users specify, performed by clicking on the corresponding item. “Delete” is to remove an IB trial.

## 7. Usage Scenarios

In this section, we take a public trajectory dataset, CROSS [6,37], to demonstrate how IBVis works on interactive analysis of trajectories. We choose 650 training trajectories with the number of sample points ranging from 6 to 19, happening at a four-way cross road intersection. The IB algorithm divides the trajectories into 12 clusters, and each of which delivers a representative pattern of traffic flow, such as straight moving and turning, as shown in Figure 6a. Since this dataset has a ground-truth, it is easy to know whether the results by IBVis are one hundred percent correct or not.

### 7.1. Investigating the Mechanism of IB

Our focus is, based on IBVis, to probe the behavior of IB clustering in each iteration, attempting to gain insight to improve IB. Here, as for the parameters used by KDE modeling, we set the number of bins as 32 and utilize the location attribute as suggested by the designers [37]. In practical applications of IB clustering, the execution efficiency and quality of clustering are important for the sake of handling complex trajectory dataset, and the use of IBVis helps to solve these issues. Generally, in each merging iteration, only two candidate trajectory clusters are combined. However, more than two candidates could be merged to speed up the clustering process. An example of the utilization of IBVis, shown in Figure 6a, indicates that the 14th pair of clusters (the clusters numbers 12 and 16) are interactively and manually merged by using the view “Merging and Backtracking”, for the 539th iteration, to obtain the same final clustering results due to the direct performing of the original IB algorithm. Moreover, the first pair of clusters (the clusters numbers 616 and 627), which are taken for merging by the original IB, in the end fall into the clustering group also containing the 14th pair of clusters. This implies that more than one pairs of clusters in an iteration could be merged, decreasing the computational burden of the IB clustering process, as has been discovered in our previous work [10]. For another example of using IBVis, as illustrated in Figure 6b, the interactive analytics methodology provided by “Merging and Backtracking” reveals that, by the merging of the 18th pair of clusters (the clusters numbers 452 and 501) for the iteration number 624, the final clustering performance is reduced to 0.9938 and 0.9867 for precision and recall measures respectively, which are all lower than 100 percent achieved by the original IB. This clearly points out that the quality control on the selection of the pair of clusters for merging is important. In addition, this also hints that one of the possible ways for the improvement of IB-based clustering is to make use of a term relevant to the clustering quality in the objective function of IB principle.

**Figure 7:**
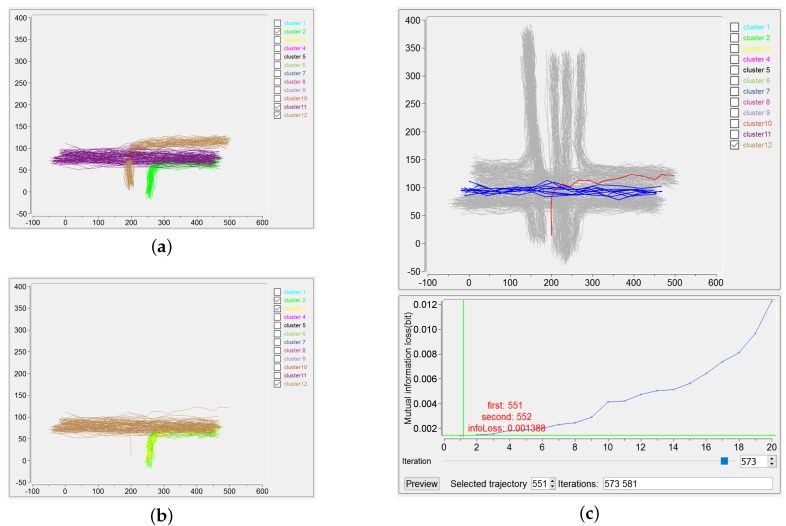
Result of IB dealing with outlier. The clustering result is worse with outlier (**b**) than with outlier (**a**); (**c**) Via IBVis, users can obtain the iteration information of the outlier. (**a**) Clustering result without outlier; (**b**) Clustering result with outlier; (**c**)Iteration information of the outlier.

The use of “Merging and Backtracking” also helps, in the procedure of IB clustering, to debug a possible clustering error due to the outliers in the trajectory dataset. It is widely known that outliers make the clustering difficult and usually outlier detection is done before the clustering [38]. Indeed, the use of effective distance metrics, such as robust Mahalanobis distance [39], could probably help to detect outliers in trajectory data. In this paper, we attempt an alternative way, namely the visualization methodology, for this issue, as described in the following example. Considering the trajectories covered by the “ground-truth” clusters 2, 11 and 12 in Figure 7a, only one trajectory of the cluster 12 is kept as an outlier (the red one in upper part of Figure 7c), and the IB clustering is performed to output the results shown in Figure 7b. The outlier and the trajectories belonging to the previous cluster 11 are incorrectly grouped to give the (new) cluster 12. Besides, the original cluster 2 is wrongly divided into the (new) clusters 2 and 3. The precision and recall for this unsatisfactory clustering are 99.60% and 91.67%, respectively, which can be obtained by checking the corresponding interface. Here, by simply picking up the outlier trajectory in Trajectory View (as illustrated by the region 3.1 in Figure 5), the ID of this trajectory (number 551) and also the iterations take this outlier for merging are all easily identified on the interface. Obviously, with the participation of users, the iteration number 573, in which the outlier is firstly included for merging, can be tracked, as demonstrated in the lower part of Figure 7c. Then users can manually choose another suitable pair of clusters for merging for this iteration, finally leading to a correct clustering result. The manageable handling of outliers for IB-based clustering offered by IBVis implies that outlier detection can be done explicitly in the procedure of clustering, this can be fulfilled by adding an item on quality control and/or by directly carrying out outlier detection in each iteration, as have been proposed in our former papers [9,11].

IBVis, as discussed above, provides an interactive manipulation upon a certain iteration to correct the IB-based clustering. In addition, the essence embedded in interactive operation suggests possible approaches to the improvement of IB clustering.

### 7.2. Exploring the Trajectory Data

Based on discovering the relationship between parameter settings and the corresponding clustering results, the trajectory data is explored and understood by the use of IBVis.

**Figure 8:**
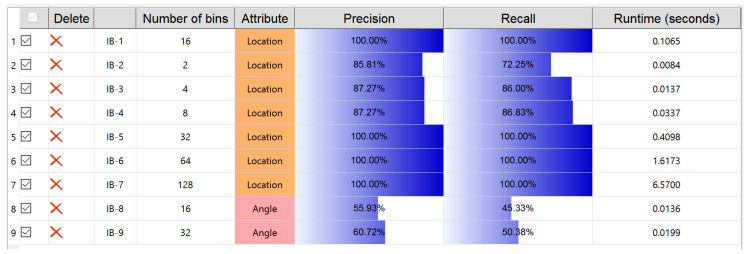
The clustering results with various parameter settings.

The different attributes taken for KDE modeling and then for IB-based clustering essentially play an important role for observing the trajectory data from diverse perspectives. As a matter of fact, to select which kind of attribute for trajectory data should be closely relevant to their application backgrounds. For example, the angle attribute is used for trajectory shape analysis [40]. As for the CROSS dataset used here, we believe that location is the most critical attribute, because all the trajectories of this dataset happen at a cross road intersection and behave according to the required traffic rules based on location. This is verified by the better performance evaluations resulted from the use of location compared with those from the use of angle: an example organization by Ranking View lists trials of IB clustering with different precision and recall values due to different attributes used (Figure 8).

The number of discrete probability bins, as evidenced in [9], is an important parameter for IB-based clustering, given a certain attribute used in KDE modeling. According to Equation (2), the mutual information loss of merging two clusters needs to compute the Jensen-Shannon divergence between two weighted PDFs. Thus, in theory, if the number of bins is large then the runtime of IB clustering becomes long, and this is obviously exemplified by the Ranking View demonstrated in Figure 8. As for the practical mastering of trajectory data, the clustering performance is of course critical. The clear presentation, by the Ranking View, on the balance between the clustering performance, the number of bins and the runtime of the algorithm undoubtedly gives an advice to understand well the trajectory data.

To sum up, IBVis benefits the exploration of trajectory dataset, based on the trials of IB clustering in different parameter settings.

## 8. User Study

We conducted a preliminary user study that gathered the feedback from users. Twenty participants were given simple description of IBVis and asked to provide a score from 0 (bad) to 10 (excellent) for each designed questions. They bear no relationship with this research and none of them had known information bottleneck (IB) principle. The 5 questions focus on different aspects of our work:

1. Interface design. How do you feel about IBVis, such as the interface layout, system architecture, colors, widgets and so on?
2. User interactions. Do you think that the interactions, such as highlight, backtracking, editing, ranking and so on, are very easy and clear to get started?
3. Stability. Can you operate IBVis smoothly? Does IBVis occur system breakdown, sudden termination or other unexpected cases?
4. Utility. Do you think IBVis is very useful and necessary to assist the understanding of IB and explore trajectory data?
5. Knowledge acquisition. How much information about the mechanism of IB do you receive from IBVis? Are you clear about how IB works for trajectory clustering now?

The marks are shown in Table 1. Most participants gave positive responses, and on average all the scores are bigger than 8. According to this preliminary user study, IBVis is acceptable.

## 9. Conclusions

Information bottleneck (IB) is good at obtaining an adaptive number of clusters, without the use of a termination threshold and distance measure. This paper has proposed and presented a simple but effective prototype, IBVis, to fulfill the visual analytics on IB-based trajectory clustering, leading to understanding and improving the iterative merging mechanism of IB and to exploring the trajectory data. The usage scenarios introduced here have shown the effectiveness of IBVis. Additionally, the user study conducted has demonstrated that IBVis is well designed, and that the rich interactive manipulations provided offer a good use experience.

In the near future, IBVis can be built as an extension on some software platforms which include a collection of machine learning algorithms and visualization tools, such as WEKA and RapidMiner, for the sake of performance comparison. Also, some powerful similarity metrics such as the so-called spatio-temporal convolution kernels [41] will be implemented in the framework of general agglomerative clustering to improve the applicability and impact of our IBVis. The divergence estimation techniques [42] could be used to directly obtain the difference between probability distributions of clusters, for the purpose of simplifying the procedure of IB-based clustering. As indicated in Section 7.1, the strategy for the iterative merging could be attempted to improve the clustering performance. In addition, we will improve IBVis to have more intuitive guidance for the new users, such as the relevant description of each function, recommend action and so on. 

## Figures and Tables

**Table 1:** User evaluation of IBVis.

User	1	2	3	4	5	6	7	8	9	10	11	12	13	14	15	16	17	18	19	20	Avg.
Interface design	8	8	7	8	10	7	8	9	9	8	10	7	9	8	9	7	8	8	9	8	8.25
User interaction	9	10	8	9	9	9	10	9	8	10	7	8	9	8	9	9	9	8	9	10	8.85
Stability	10	8	9	7	9	8	8	9	9	9	8	9	9	7	7	9	9	8	8	9	8.45
Utility	9	8	9	9	8	8	9	8	8	9	9	7	8	9	8	7	8	8	10	8	8.55
Knowledge acquisition	7	8	8	8	9	9	8	8	8	8	7	9	7	9	8	8	7	8	8	9	8.05
